# Dissociable effects of LSD and MDMA on striato-cortical connectivity in healthy subjects

**DOI:** 10.1038/s41386-025-02270-5

**Published:** 2025-10-31

**Authors:** Natalie Ertl, Imran Ashraf, Lisa Azizi, Leor Roseman, David Erritzoe, David J. Nutt, Robin L. Carhart-Harris, Matthew B. Wall

**Affiliations:** 1 Perceptive, Hammersmith Hospital, London, UK; 2 Faculty of Medicine, Imperial College London, London, UK; 3 Centre for Psychedelic Research, Division of Psychiatry, Imperial College London, London, UK; 4 Department of Psychology, University of Exeter, London, UK; 5 Carhart-Harris Lab, Dept. of Neurology, University of California San Francisco, San Francisco, USA

**Keywords:** Neuroscience, Reward

## Abstract

Lysergic acid diethylamide (LSD) and 3,4-Methylenedioxymethamphetamine (MDMA) are widely used psychoactive drugs and their potential use in psychiatric medicine is currently generating interest. The mechanism by which these drugs may assist recovery in various disorders such as addiction and post-traumatic stress disorder (PTSD) is still not well understood. Most investigations of the effects of these drugs on brain activity have focused on cortical resting-state networks, however the striatum is a key reward and motivation hub of the brain and aberrant striatal processing may be part of the pathophysiology of these disorders. Consequently, we investigated striatal connectivity following acute MDMA and LSD administration. Resting-state fMRI (rs-fMRI) data were acquired from two separate previous studies, and seed-voxel functional connectivity analyses were used with the striatum subdivided into three seed regions: the associative, limbic, and sensorimotor striatum. Within-network connectivity was measured using group mean network maps and whole-brain connectivity (seed-to-voxel) was also examined. Neither MDMA nor LSD significantly changed within-network connectivity of any of the three striatal seed regions. However, striatal connectivity with other brain regions was significantly altered with both MDMA and LSD. Most notably, MDMA reduced connectivity between the limbic striatum and the amygdala, while LSD increased connectivity between the associative striatum and the frontal, sensorimotor, and visual cortices. Changes in connectivity were mostly observed outside the standard striatal networks, consistent with previous findings that psychedelics reduce network modularity or between-network segregation and increase connectivity across standard networks.

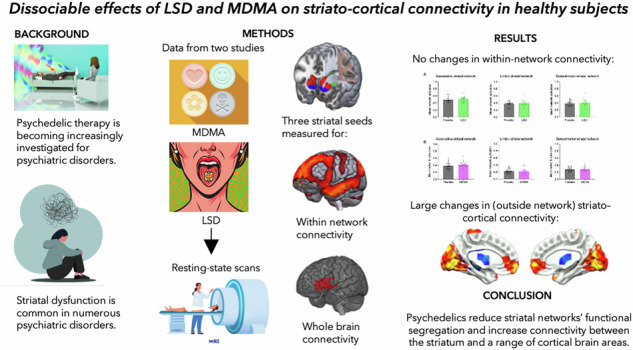

## Introduction

In recent years, the intersection between recreational substances and psychiatric medicine has garnered significant attention, particularly in the case of psychedelics such as Lysergic acid diethylamide (LSD), psilocybin, and N,N-Dimethyltryptamine (DMT), as well as the entactogen 3,4-Methylenedioxymethamphetamine (MDMA), sometimes classed as an A-typical psychedelic. The use of psychedelics as medicines has been practised by different groups around the world for thousands of years [1], however their recreational use and the consequent legal restrictions has held back scientific research and clinical applications for decades [2–4].

Classic psychedelics are agonists at serotonin 5-hydroxytryptamine (5-HT)_2A_ receptors [5], causing profound cognitive disturbances and mood-altering effects [6]. LSD is a classic psychedelic with a particularly long duration of up to 12 h [7]. Its powerful impact on perception and consciousness has made it a subject of extensive research [3, 8–10] with users commonly reporting enhanced sensory perceptions, vivid hallucinations, and alterations in the sense of self [11]. LSD was widely used clinically in the first wave of psychedelic research in the 1950s and 1960s [12] for numerous disorders including alcohol addiction [3, 13, 14], depression [15], and management of pain and anxiety in terminal illness [16, 17]. Its extended pharmacokinetics have made it a less popular choice for modern clinical research, with most studies preferring shorter-acting compounds such as psilocybin, however recent studies have shown promising results for treating anxiety [18] and depression [19].

Early neuroimaging studies with LSD showed increased blood flow and connectivity with the visual cortex under LSD, which correlated with subjective reports of complex visual imagery [8]. Other studies have identified increases in global functional connectivity, and these effects have correlated with LSD induced ego dissolution [20], and other subjective effects (particularly in the somatomotor network [21]). Functional connectivity changes also interact with music listening and correlate with eyes-closed visual imagery [22]. Graph theory analyses have demonstrated that LSD produces more network functional complexity, increases randomness, and decreases segregation of functional brain networks [23]. In common with other classic psychedelics such as psilocybin [24] and DMT [25], LSD therefore has a wide-scale disruptive effect on cortical brain networks, and tends to increase network integration and decrease segregation/modularity.

MDMA (sometimes now called midomafetamine) is an entactogen that acts as a monoamine releaser and a reuptake transporter inhibitor [26]. This makes it more akin to amphetamines [27], with only mild hallucinogenic properties. Like classic psychedelics, its primary impact is on serotonin (5-HT), however MDMA’s stimulant-like qualities comes from its lesser effects on dopamine and noradrenaline [28]. Unlike typical stimulants, it functions as an empathogen, enhancing social-emotional processing [29]. MDMA was researched in therapeutic settings in the late 1970s and early 1980s, predominantly for couples therapy and trauma management due to its empathogenic properties [30]. The neuroimaging research into MDMA’s effects on the brain are limited in comparison to LSD, however task-fMRI studies have suggested MDMA increases frontoparietal activation in the Go/No-Go task [31], may alter the amygdala responses to emotional faces [32], (though see [33] for conflicting results) and has effects on the response to emotional autobiographical memories [34]. Functional connectivity analyses show that MDMA likely does not have the wide-spread disruptive effect on resting-state networks characteristic of classic psychedelics [35] but seed-based analyses have implicated connectivity changes in the medial temporal lobe (amygdala, hippocampus) and insula as being implicated in the action of MDMA [36, 37]. Despite the pharmacological differences between the classic psychedelic LSD and the empathogen MDMA, both compounds produce acute alterations in emotional processing, social connectedness, and consciousness [38, 39], which have been associated with therapeutic effects in psychiatric disorders involving striatal dysfunction. Like LSD, MDMA is currently being investigated for its potential therapeutic use, for a number of disorders [40].

Most existing neuroimaging research on LSD and MDMA has primarily focused on the effects of the drugs on cortical network systems, or sub-cortical regions such as the thalamus [21, 41–43]. However, many of the psychiatric conditions for which these substances are being investigated, such as PTSD, are thought to be rooted in striatal dysfunctions [44, 45]. Cortical-Striatal-Thalamic-Cortical (CSTC) loops are a key concept in a number of other disorders [46] such as Obsessive Compulsive Disorder (OCD) [47] and Attention Deficit Hyperactivity Disorder (ADHD) [48]. The striatum is highly innervated by the substantia-nigra and the ventral tegmental area, receiving a substantial proportion (60–80% [49]) of the dopamine neurons in the brain and this makes it a key region in the neurocircuitry of addiction [50]. The striatum, a collection of subcortical regions, can be functionally divided [51–53] into three key areas: the limbic striatum, which includes the nucleus accumbens and the inferior portion of the putamen; the sensorimotor striatum, encompassing the superior portion of the putamen and the tip of the caudate; and the associative striatum, which covers the remaining parts of the caudate and putamen. These subdivisions are critical for various functions: the limbic striatum is involved in motivation, reinforcement, and emotion, the sensorimotor striatum in habit formation and motor learning, and the associative striatum in decision-making and cognitive control [51].

The distinct functional subdivisions are each involved in different cortico-striatal loops, and so play a significant role in many psychiatric disorders that might be amenable to treatment with either LSD or MDMA. Cortico-striatal-thalamic circuits also play a key role in one model of the effect of psychedelic drugs [54–56], and examining striatal effects of these compounds is therefore a useful test of this model. Comparative analysis of LSD (classic psychedelic) and MDMA (‘atypical’ psychedelic/empathogen) provides a unique opportunity to examine whether distinct pharmacological mechanisms leading to altered states of consciousness share common effects on cortico-striatal circuitry, or whether there are meaningful differences. For these reasons, we investigated changes in striatal connectivity using resting-state fMRI data from acute challenge studies with these two compounds. Our hypothesis was that both compounds would significantly affect striatal connectivity, but that (based on previous work examining cortical networks with psilocybin and MDMA [35]) the effects of LSD would likely be more prominent and wide-spread.

## Methods

The current data are reanalyses of previously published research on LSD [8] and MDMA [36], please see these publications for the full study protocol.

### Ethics

Both studies were approved by the National Research Ethics Service West London Research Ethics Committee, all subjects provided written informed consent, and the studies were conducted in accordance with good clinical practice guidelines. For more information, please refer to the original manuscripts [8, 36].

### Study design

#### LSD

The LSD study used a single-blind (participant blinding only), balanced-randomised design. 75 µg LSD or placebo was administered via identical IV solutions (more information in Table 2). Participants attended two scanning days at least two weeks apart to minimise carry-over effects, previous studies have reported a variable half-life of 75 µg LSD, suggesting it ranges from 2.2 to 4.3 h [57]. Patients lay inside a mock MRI scanner for 60 min and were encouraged to relax with their eyes closed, in order to acclimatise the participants to the MRI scanning environment and to reduce any potential anxiety later in the trial. Following this, subjects were moved to the real MRI scanner for a set of scans which included: a structural scan, arterial spin labelling (ASL) and resting-state fMRI. Two resting-state scans were completed per treatment session, one at 70 mins post dose and the next at 90 min, separated by a music listening scan. Participants were encouraged to lie with their eyes closed. After the MRI scanning, there was a break of approximately 35 min, after which magnetoencephalography (MEG) scanning was performed (not reported here). Once the subjective effects of LSD had sufficiently subsided, the study psychiatrist assessed the participant’s suitability for discharge.

#### MDMA

The MDMA study was a double-blind, placebo controlled, within-subjects, randomised-controlled trial. The participants underwent two scanning sessions, seven days apart, identical other than the administration of either MDMA or placebo in a randomised order. Both participants and researchers were blinded to the substance administered. The administration route was via identical capsules (see Table 2 for more details). Participants underwent two resting-state scans per visit (four in total). The first scan took place approximately 60 min after drug administration and the second at approximately 113 min post drug administration. Participants were encouraged to relax in the MRI machine with their eyes closed.

#### Participants

Participant characteristics can be found in Table 1.

**Table 1:** Participant characteristics for LSD and MDMA study and main inclusion criteria.

	LSD, *N* = 16	MDMA, *N* = 22
**Age (years) mean, range**	29.93 ± 7.41	35 ± 9.8
**Female N (%)**	4 (23.5%)	6 (27%)
**Male N (%)**	13 (76.5%)	16 (73%)
**Inclusion criteria**	• >21 years old • Previous experience with MDMA/Psychedelics • No current, historical or immediate family members with psychiatric disorders • No drug or alcohol dependencies • No MRI contradictions	
	• No MDMA or drug use for the past 7 days	• No psychedelic drug use for the past 6 weeks
**Exclusions for head-motion (HM) and other scan quality violations**	Starting *N* = 19 -2 for HM, -1 for other scan artifact Final *N* = 16	Starting *N* = 25 -3 for HM Final *N* = 22

#### Drug administration

LSD was administered intravenously. 75 µg LSD in 10 ml saline was administered over two minutes, followed by saline wash. The placebo was 10 ml saline, administered over two minutes, followed by saline wash. MDMA was administered orally. The dose was a 100 mg capsule of MDMA-HCL, while the placebo was 100 mg of vitamin C.

#### MRI acquisition

Anatomical T1 weighted images were acquired in both scans. The details of the functional acquisitions are listed in Table 2.

**Table 2:** Summary of MRI acquisition of the two studies.

*Functional resting-state scans*	LSD	MDMA
**Scanner**	3 T GE HDx system	3 T Siemens Tim Trio scanner
**Sequence**	T2*-weighted echo-planar images (EPI)	T2*-weighted echo-planar images (EPI)
**Repetition time (TR)**	2 s	2 s
**Echo time (TE)**	35 ms	31 ms
**Isotropic voxel size**	3.4 mm	3 mm
**Slice acceleration factor**	SENSE = 2	GRAPPA = 2
**Flip angle (degrees)**	90	80
**Axial slices per TR**	35	36
**Total scan time**	7 min 20 s	6 min

#### fMRI analysis

All analyses were conducted using FMRIB Software Library (FSL) 6.0, and broadly following an approach used in previous work [58–63]. The data was pre-processed using standard procedures: head-motion correction using MCFLIRT, non-linear registration to a standard template (MNI152), high-pass temporal filtering (0.01 Hz), and spatial smoothing with a 6 mm FWHM (full-width, half-maximum) Gaussian kernel. At this stage head motion parameters were examined, and each scan was assessed for mean framewise displacement >0.5 mm or maximum displacement >3 mm in any direction, if these measures were exceeded on either session, then the participant was excluded. Framewise displacement measures were derived using the fsl_motion_outliers function and paired *t* tests on these values were conducted between the placebo and drug conditions, in order to check for head-movement differences between the treatments.

The anatomical data were parcellated using FMRIB’s automated segmentation tool (FAST), producing white matter (WM) and cerebrospinal fluid (CSF) segmentations. These were coregistered into individual subjects’ functional data space and thresholded at 0.5. The mean time-series from these parcellations were extracted to be used as nuisance regressors in the model [64]. Description of an alternative denoising method and associated results can be found in the supplementary materials.

Seed-voxel analysis was used to assess striatal functional connectivity; this approach assumes that voxels which are activating in a similar manner to that of the seed are likely functionally connected. The striatal seeds selected followed the original parcellation by [51], using the atlas provided by [52], and are shown in Supplementary Fig. 1. Each seed (in MNI152 space) was registered to the participants structural and then functional scan, and the individual seed-region masks in functional space were then thresholded at 0.5. The time-series from the participants’ seed regions was then extracted and this was used as the regressor of interest in the model. The WM and CSF regressors, along with an extended set of head-motion parameters (set of 24 head-motion parameters, including six original regressors: three translations, three rotations, plus temporal derivatives and quadratic versions of the original six regressors) were also added to the model.

**Fig. 1:**
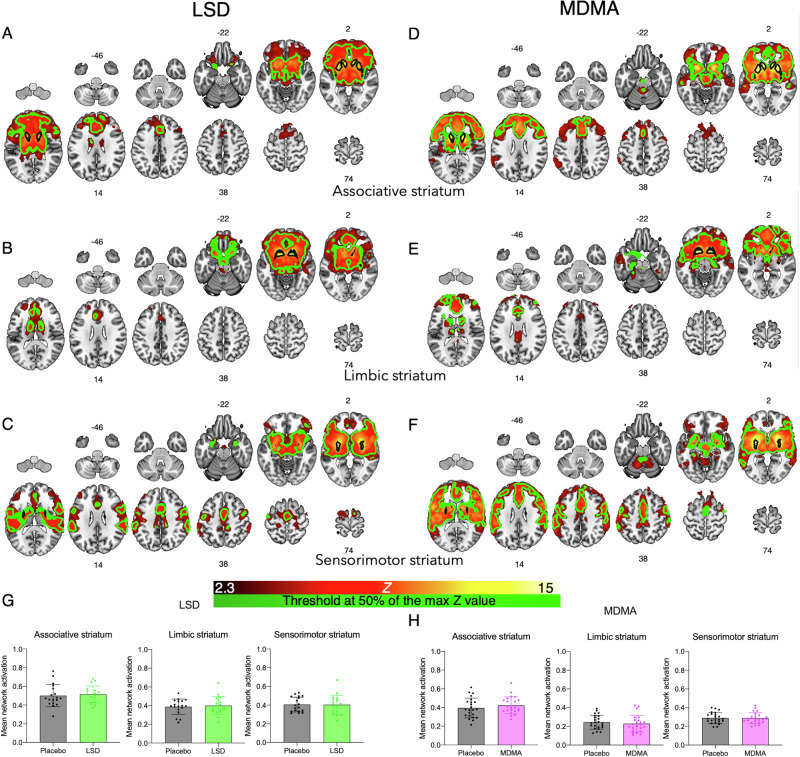
Network ROIs derived from mean network maps. Mean network maps averaging placebo and LSD with the **A** associative striatal seed, **B** limbic striatal seed, **C** sensorimotor striatal seed; and placebo and MDMA with the **D** associative striatal seed, **E** limbic striatal seed, **F** sensorimotor striatal seed. LSD *N* = 16, MDMA *N* = 22, results are cluster corrected (*Z* = 2.3) and thresholded *P* < 0.05. Overlayed black outlines are the original seed regions. Overlayed in green outline is the region thresholded at 50% of the max Z score mask used to derive the network ROI in the subsequent analyses. Numbers represent MNI slices. **G, H** No changes in within-network connectivity were identified in any of the striatal networks with **G** LSD (green) or **H** MDMA (pink) administration relative to placebo, LSD *N* = 16, MDMA *N* = 22, error bars show SEM.

Next fixed effects mid-level analyses were run to average the results from the first and second resting state scan for each treatment session of each participant. These mid-level analyses were then used in the higher-level models. All group-level analyses were conducted using FMRIB’s local analysis of mixed effects (FLAME-1) method, employing cluster-level thresholding (*Z* = 2.3, *p* < 0.05) to account for multiple comparisons. This threshold provides appropriate control for Type I errors when used with FSL’s FLAME-1 model [65], while also maintaining sensitivity and thereby minimising Type II errors [66, 67]. Initially, as a validation step, a group mean (all subjects, drug and placebo scans – for LSD and MDMA separately) analysis was performed to produce overall networks for each seed region, these networks were corroborated against previous studies [58–60, 68]. To investigate overall within-network connectivity, these derived networks were thresholded at 50% of their maximum Z values and binarised to produce network masks (see Fig. 1). A mean connectivity parameter estimate for each participant for each drug condition was extracted from these masks and a paired *t* test was conducted to investigate significant differences in within-network connectivity between placebo and drug scans.

Next, to test for changes in connectivity with each network and the rest of the brain, seed-voxel analysis was performed with a within-subjects effects model. This showed regions of the brain which were relatively more or less connected with the seed regions in the active drug compared to placebo scans.

Finally, correlations were conducted to assess if changes in striatal connectivity are associated with ratings of subjective drug effects. The connectivity parameter estimates from the drug condition (LSD/MDMA) from the within-connectivity maps were correlated to the subjective drug ratings using Pearson’s correlation in a correlation matrix and corrected for multiple comparisons.

## Results

### Head-motion

Two participants were excluded for excessive head-motion (>3 mm max frame-wise displacement) and one for a separate artifact, from the LSD analysis; once they were removed from further analysis, a paired *t* test showed a significant difference in mean framewise displacement between the placebo (mean FD = 0.094, SD = 0.041) and LSD (mean FD = 0.143, SD = 0.062) condition *t*[31] = 4.58, *P* < 0.0001. Three participants were excluded from the MDMA analysis due to excessive head-motion, there was also a significant difference in mean head movement between the placebo (mean FD = 0.111, SD = 0.042) and MDMA (mean FD = 0.135, SD = 0.072) conditions *t*[43] = 2.29, *P* = 0.026.

### Within-network connectivity

Striatal connectivity networks were derived from averaging across the placebo and drug conditions for each seed region. These networks closely match previous work using different subject cohorts [58, 68], with the associative striatum characteristically showing connectivity with the frontal lobe, the limbic striatum being strongly connected with medial-temporal-lobe regions, and the sensorimotor striatum showing strong connectivity with the motor cortex. Similar patterns of activation were seen in the LSD (Fig. 1A–C) and MDMA groups (Fig. 1D–F), thereby validating this seed-voxel approach and the analysis procedures. The network ROI (derived from thresholding at 50% of the maximum Z value), is also shown on Fig. 1A–F as a green outline. Changes in connectivity from within the derived network ROIs is shown in Fig. 1G, H. No significant changes in within-network connectivity were found in any of the striatal seeds with LSD or MDMA administration. Group average results of the placebo and drug conditions are separately shown in Supplementary Fig. 2, MNI coordinates in Supplementary Table 1, 2.

### Seed-voxel (whole-brain) analysis

Next, striatal network connectivity with the rest of the brain (whole-brain, seed-to-voxel analyses) was investigated.

### LSD

**Fig. 2:**
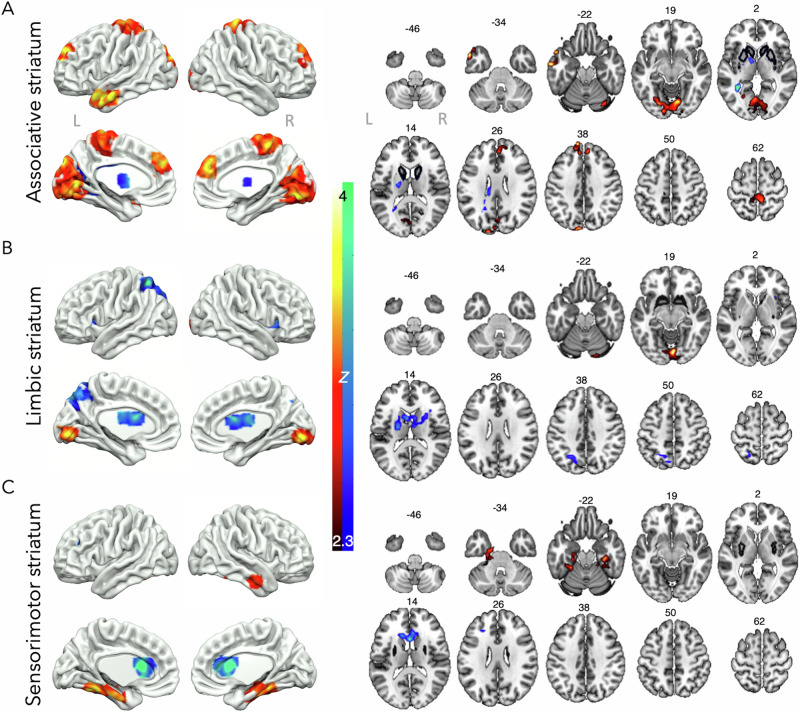
Striatal connectivity changes with LSD. Acute LSD significantly increased (red/yellow) and decreased (blue/green) connectivity between the **A** associative striatum, **B** limbic striatum, and **C** sensorimotor striatum and areas in the rest of the brain, *N* = 16, results are cluster corrected and thresholded at *Z* = 2.3, *p* < 0.05, original seed regions shown in black on axial slices, MNI slices labelled.

Acute LSD administration altered whole-brain connectivity with all three of the striatal seeds investigated (Fig. 2A–C). Increases in connectivity were observed between the associative striatum and a large cluster spanning the visual cortex, as well as the bilateral secondary sensorimotor cortex and medial frontal regions. Decreased connectivity was observed with the tail of the left putamen (part of the sensorimotor striatum) extending into the thalamus (Fig. 2A). The limbic striatum significantly increased its connectivity with the left frontal orbital cortex and an area around the inferior occipital cortex from the occipital pole extending into the lingual gyrus. Decreased connectivity was observed with an area in the extending from the inferior parietal cortex into the superior lateral occipital cortex (Fig. 2B). Finally, connectivity with the sensorimotor striatum and an area in the bilateral parahippocampus extending into the temporal cortices and an area around the precuneus extended into the intracalcarine cortex significantly increased under acute LSD. A decrease was observed around the rostral anterior cingulate and the left inferior frontal gyrus (Fig. 2C). Cluster coordinates can be found in Supplementary Table 3, with supplementary slices in Supplementary Fig. 3.

### MDMA

**Fig. 3:**
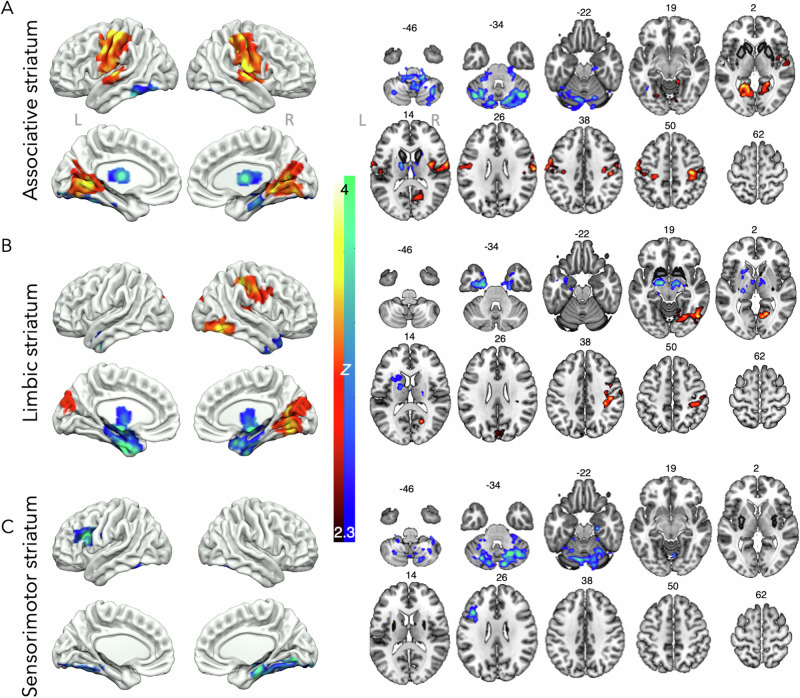
Striatal connectivity changes with MDMA. Acute MDMA significantly increased (red/yellow) and decreased (blue/green) connectivity between the **A** associative striatum, **B** limbic striatum, and **C** sensorimotor striatum and areas in the rest of the brain. Results are cluster corrected and thresholded at *Z* = 2.3, *p* < 0.05, *N* = 22, original seed regions shown in black on axial slices. MNI slices labelled.

MDMA also significantly altered brain connectivity with all three of the striatal networks investigated (Fig. 3A–C). Under acute MDMA treatment, associative striatum connectivity with the sensorimotor cortex and lingual gyrus was significantly increased, whereas connectivity with the cerebellum was significantly reduced (Fig. 3A). Connectivity between the limbic striatum and the sensorimotor cortex as well as higher order visual areas such as the lingual and intracalcarine cortex was also increased, while connectivity between the limbic striatum and the thalamus, amygdala, and hippocampus was significantly reduced under acute MDMA administration (Fig. 3B). Finally, connectivity between the sensorimotor striatum and the inferior frontal gyrus and cerebellum was reduced under acute MDMA administration (Fig. 3C). Cluster coordinates can be found in Supplementary Table 4, with supplementary slices in Supplementary Fig. 4.

### Correlation analysis

No significant correlations between network connectivity and subjective ratings of acute drug effects under acute LSD or MDMA were uncovered. Refer to Supplementary Tables 1 and 2 for details.

## Discussion

The results show that neither LSD nor MDMA have strong effects on the broad measure of within-network connectivity relative to placebo. However, both drugs produced marked changes in connectivity with brain regions outside of the standard striatal networks. This aligns with previous research suggesting that psychedelics may reduce brain modularity [69] and increase global functional connectivity [21, 70]. When modularity is reduced, overall network structure is degraded, and brain regions/networks become more interconnected and communicate more with other regions/networks that they typically do not synchronise with. This effect of psychedelic and empathogenic drugs may allow for novel patterns of thought and perception to emerge, and be related to their clinical effects [71]. Previous work has shown strong effects of the classic psychedelic psilocybin on cortical between-network connectivity, with relatively few effects of MDMA [35]. In contrast, here we show that MDMA may actually have reasonably comparable effects to the classic psychedelic LSD on striatal networks, although with important distinctions as well. These findings are consistent with the known projection targets of these functionally distinct striatal subregions and can be interpreted within the framework of cortico-striato-thalamo-cortical (CSTC) loops - anatomically and functionally segregated circuits that support cognitive (associative), affective (limbic), and sensorimotor processes. Dysfunctions in CSTC loops are implicated in a number of psychiatric disorders [46] and CSTC functions have been proposed to play a major role in mechanisms of consciousness [72, 73]. One major model of psychedelic drug action proposes the cortico-striatal-thalamic pathways as central to the disruptions of perception and cognition seen with these drugs [54, 55], and the current data provide more evidence to support this perspective.

### LSD

Of the three striatal seed regions investigated under acute LSD administration, the associative striatum showed greatest changes in connectivity, with significant increases in the lingual gyrus, sensorimotor cortex, and frontal poles, as well as a small reduction in connectivity with the thalamus. The associative striatum has functions in cognitive integration and memory processes [74]. The lingual gyrus and medial occipital lobe are involved in visual processing [75] and increased connectivity with these areas under LSD may reflect the intense visual hallucinations often reported with an LSD experience. Increases between striatal and visual regions have been previously identified [76] and further more researchers [77] have recently emphasised that the unique effects of psychedelics on visual function are an important (and perhaps, overlooked) part of their phenomenology and that low-level sensory effects are likely to significantly influence higher-level brain systems. The finding here that the (‘cognitive’) associative striatum’s connectivity with the visual cortex is strongly increased with LSD supports this thesis. Under acute LSD the associative striatum increased connectivity with the sensorimotor cortex, possibly enabling deeper and more meaningful sensory-emotional experiences and a heightened sense of feeling connected to surroundings or others [78]. Additionally, increased functional connectivity was observed between the associative striatum and the frontal poles. The frontal cortex is involved in executive control and cognition [79], so this change in connectivity may reflect the alterations in cognitive function often experienced under acute LSD, for example increased reinforcement learning ability [80].

Relative to the associative striatum, LSD caused less disruption in limbic striatal connectivity. The limbic striatum contains the nucleus accumbens which has a prominent role in reward and consequently addiction; the limited changes in connectivity observed with these addiction-relevant areas is consonant with the lack of addictive properties seen with classic psychedelics such as LSD [81, 82]. Moderate changes in limbic striatal connectivity were observed with the visual cortex again emphasising the altered connectivity of the visual cortex during an LSD trip likely leading to hallucinations or altered visual perception.

The sensorimotor striatum is involved in the integration of sensory information and motor planning and execution [83]. It receives inputs from various sensory modalities, processes this information, and is involved in the coordination and execution of voluntary movements [84]. Additionally, the sensorimotor striatum is essential for habit formation, procedural learning, and motor skill acquisition [85]. Under LSD administration, increased connectivity was observed between the sensorimotor striatum and regions in the temporal lobes including the parahippocampus. This may be significant since the parahippocampus is involved in spatial memory [86]. LSD also reduced connectivity between the sensorimotor striatum and the left inferior frontal gyrus, this reduction in connectivity with the brain’s inhibitory control centre under acute psychedelics may be reflective of the feelings of euphoria experienced under psychedelics as well as the sense of dissociation or altered bodily awareness. Users of psychedelics often report feelings of mental lightness, disembodiment, or a disconnection from physical boundaries [35].

### MDMA

Under acute MDMA administration, increased connectivity between both the associative and limbic striatum with the sensorimotor cortex was observed. The associative striatum is pivotal in cognitive processing [74], while the limbic striatum also has a role in emotional processing as well as reward/motivational systems [87], and so this increased connectivity with the sensory integration brain regions could reflect the enhanced emotional experiences under MDMA [88]. Interestingly, reduced connectivity was observed between both the associative and sensorimotor striatum with an area in the cerebellum under acute MDMA. The cerebellum is also involved in motor coordination and reduced connectivity with this area may reflect the changes in balance and motor coordination seen with MDMA [89]. Specifically, the cluster of deactivation spanned the cerebellar vermis bilaterally. Since the vermis is a key area in oculomotor control [90], decreased connectivity with this area could represent the reduced eye-motor control and increase in saccadic movements sometimes experienced with MDMA users [91].

Limbic striatal connectivity with the amygdala and parahippocampus was downregulated under acute MDMA administration. MDMA’s inhibitory effect on the amygdala is particularly noteworthy, given the amygdala’s central role in fear response and fear memory formation [92]. Individuals with PTSD often have hyperactivity in the amygdala [93] and have exaggerated fear responses, hindering their ability to engage effectively in therapy [94]. By attenuating the amygdala’s response, MDMA could enable individuals to access their trauma memories in a controlled manner, facilitating therapeutic interventions without triggering excessive fight or flight reactions. These findings are consistent with previous work showing changes in amygdala connectivity are related to symptom improvement in PTSD patients treated with MDMA therapy [95]. Moreover, evidence suggests that survivors of the 7^th^ October attacks in Israel who had taken MDMA showed some protection against the development of PTSD compared to survivors who had not taken MDMA [96], suggesting MDMA may actually prevent the traumatic memories from forming in the same way, potentially through a relative functional disconnection involving the amygdala.

Acute MDMA administration led to a decrease in connectivity between the sensorimotor striatum and a region around the left inferior frontal gyrus and a region in the cerebellum. Specifically, this cerebellar deactivation cluster spanned the right vermis and crossed into the right Crus I and a small area in the left vermis, involved in motor and oculomotor control [90]. The left inferior frontal gyrus is thought to be part of the brain’s inhibitory control network [97]. Connectivity between the inferior frontal gyrus and sensorimotor striatum may play a role in suppressing unwanted or inappropriate motor responses. A reduction in connectivity between these regions might imply a temporary disruption in the brain’s ability to regulate and inhibit certain movements.

### Clinical Implications

Altered striato-cortical connectivity is observed in addiction and affects cognition, working memory, attention, and decision-making [98]. LSD may enhance connectivity between the associative striatum and frontal cortex, which could improve cognition and self-control, and so reduce impulsivity. This may suggest potential in treatment for addiction [99], although further research is needed. MDMA, known for its effects on emotional openness and enhancing social interactions, may reduce inhibitory control, allowing emotional release. Its increased connectivity between the limbic striatum and sensory cortex may help process trauma, supporting its potential in PTSD treatment [100]. However, scientific rigour in MDMA research needs improvement [101].

### Comparing typical and atypical psychedelics

LSD and MDMA differ substantially in their neuropharmacological profiles and some aspects of the subjective experience, yet they both possess ‘mind-manifesting’ properties and therapeutic potential [12, 40, 99]. Our study reveals overlapping patterns of increased connectivity from both the associative and limbic striatum to posterior cortical regions, including the lingual gyrus and precuneus. However, connectivity changes from the sensorimotor striatum showed little commonality. MDMA was associated with decreased connectivity to the cerebellum, while LSD increased connectivity in a small cluster in the posterior temporal cortex. Importantly, neither drug produced significant changes in within-network connectivity as defined by the striatal seed-based network maps. This absence of within-striatal-network alterations with MDMA and LSD may relate to the non-addictive properties of both compounds, contrasting with findings from acute heroin administration in dependent individuals, where increased limbic network connectivity correlated with the subjective ‘rush’ experience [102]. A recent study directly comparing LSD and MDMA with a cortical network framework found that LSD induced broader between-network reconfiguration particularly between the auditory-sensorimotor and dorsal attention networks, while MDMA reduced within-auditory-sensorimotor-network connectivity and LSD reduced default mode network connectivity [103]. Although our approach focused on subcortical–cortical connectivity rather than cortical resting-state networks, the differing profiles of LSD and MDMA across both studies point to more extensive systems-level reorganisation under LSD relative to MDMA, particularly in networks involved in attention and sensory integration.

### Strengths and limitations

This study is among the first to specifically investigate changes in striatal connectivity under classic and atypical psychedelics. The placebo-controlled design accounts for between subject differences, allowing for a more accurate detection of the effects of the drugs on striatal connectivity to be uncovered. Despite rigorous correction for head-motion and exclusion criteria, significant differences in head-motion between drug and placebo sessions remain, meaning that head-motion cannot be ruled out as a potential confounder. Mitigating against this interpretation though are the null findings on within-network connectivity, where head-motion might also be expected to have an effect (if it was a potentially serious confounder). While these are re-analyses of some of the largest LSD and MDMA research studies to date, they are both still relatively small sample sizes in fMRI research. Hopefully as psychedelic research becomes more mainstream and legal restrictions are conceivably relaxed, future studies should aim to replicate the present analysis with larger sample sizes. Moreover, the distribution of males to females in both studies is unbalanced. Research is currently suggesting that psychedelics may have particular benefits to women, which have yet to be studied in a clinical setting [104, 105]. Future studies should aim to make recruitment for trials such as these more appealing to female volunteers.

## Conclusion

In conclusion, our study examined the neurobiological effects of one classic, and one atypical (LSD and MDMA) psychedelic, revealing nuanced alterations in striatal connectivity and broader brain networks. Notably, MDMA exhibited a reduction in connectivity between the limbic striatum and amygdala, suggesting a functional mechanism for the therapeutic intervention in PTSD. Meanwhile, LSD increased connectivity between the associative striatum and frontal and visual cortices, shedding light on the drug’s impact on sensory integration, creativity, and altered cognition. The impact of this data suggests LSD may have potential as a therapy in reward-based disorders where the brain states need to be remapped, such as addictions [99], obsessive compulsive disorder [106], or hypoactive sexual desire disorder [107]. These findings may underscore the mechanism by which psychedelic medicines enhance emotional intelligence, empathy, and creative expression. By unravelling the neural intricacies of MDMA and LSD on striatal connectivity, our research adds to the body of literature which will hopefully enable targeted therapeutic applications, promising hope for individuals grappling with addiction, trauma, and emotional challenges.

## Supplementary information

Supplementary material

Supplementary Table 6

## Data Availability

LSD data available at https://openneuro.org/datasets/ds003059/versions/1.0.0. MDMA data available upon reasonable request.
