# Supplementary material for: Dissociable effects of LSD and MDMA on striato-cortical connectivity in healthy subjects
Source: Neuropsychopharmacology. 2025 Oct 31;51(2):412–21. doi: 10.1038/s41386-025-02270-5 (PMC12708869; doi:10.1038/s41386-025-02270-5)
Supplement: Supplementary file 1 — Supplementary material [file 41386_2025_2270_MOESM1_ESM.docx]

Supplementary Material

Table of Contents

[Supplementary Figure 1. 2](#_Toc210037973)

[Supplementary Figure 2. 3](#_Toc210037974)

[Supplementary Table 1. 4](#_Toc210037975)

[Supplementary Table 2 5](#_Toc210037976)

[Supplementary Figure 3. 6](#_Toc210037977)

[Supplementary Table 3 7](#_Toc210037978)

[Supplementary Figure 4. 8](#_Toc210037979)

[Supplementary Table 4 9](#_Toc210037980)

[Supplementary Table 5. 10](#_Toc210037981)

[Supplementary Table 6. 12](#_Toc210037982)

[Alternative denoising 12](#_Toc210037983)

[Supplementary Figure 5 13](#_Toc210037984)

[Supplementary Figure 6 14](#_Toc210037985)


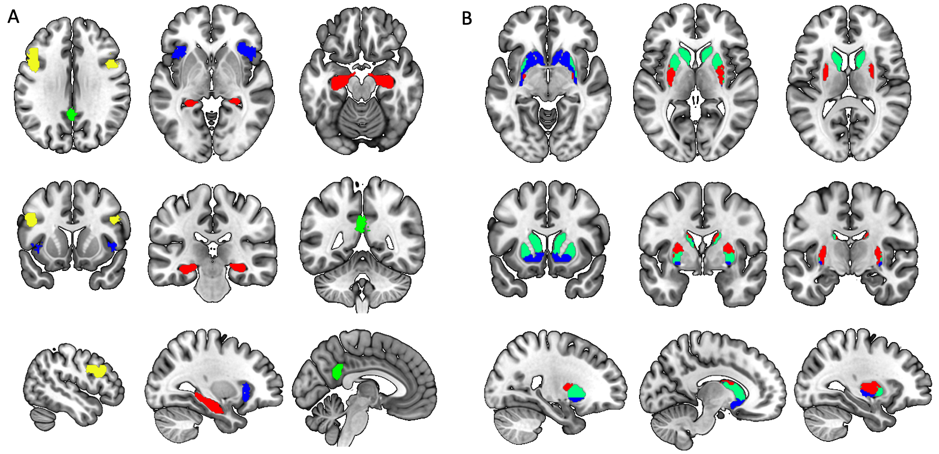


Supplementary Figure 1. The striatal networks were defined using the associative (green), limbic (blue) and sensorimotor striatum (red).


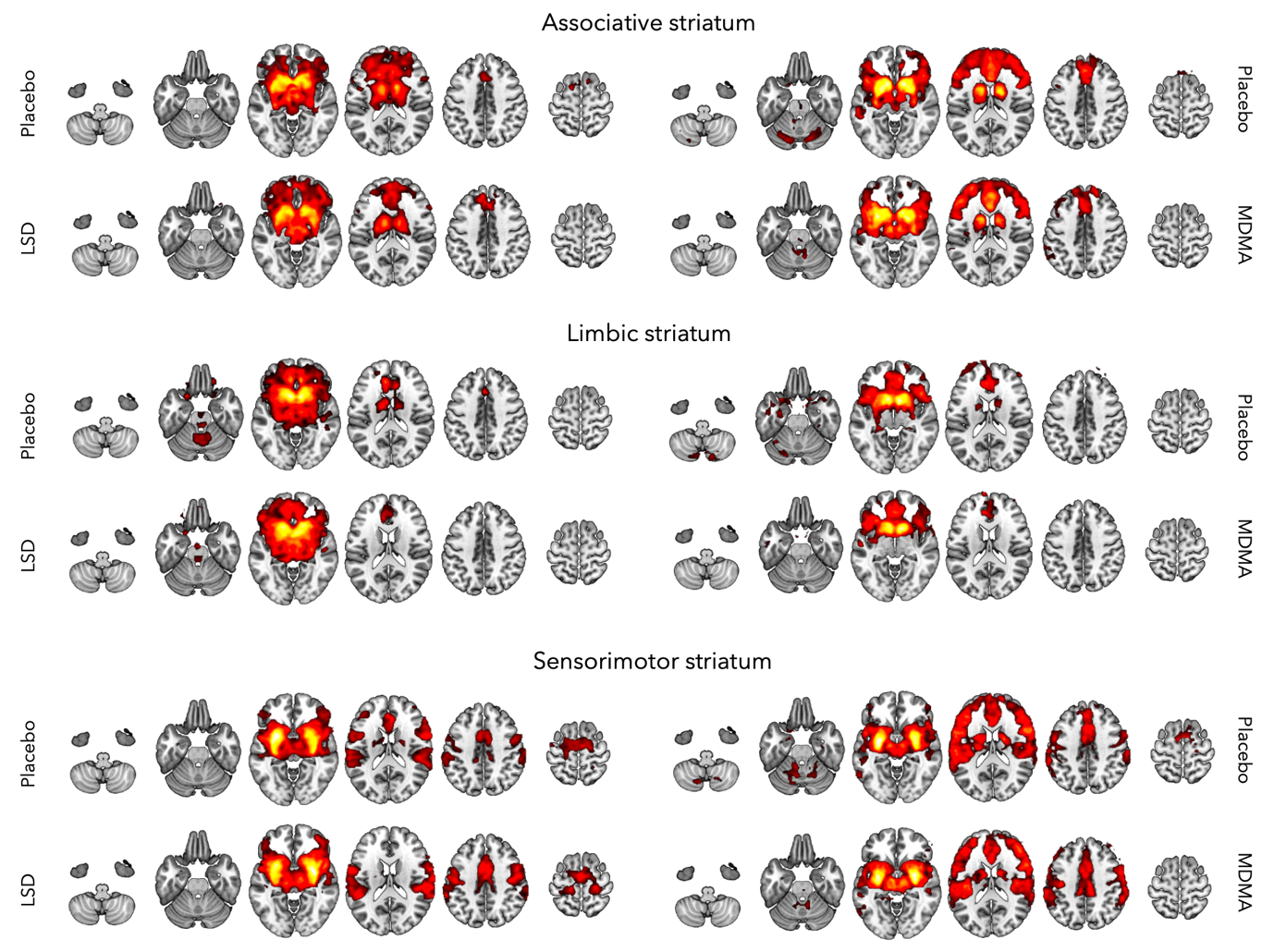
 Supplementary Figure 2. Group average of placebo and drug conditions separately. LSD N=16, MDMA N=22, results are cluster corrected (Z=2.3) and thresholded P<0.05.


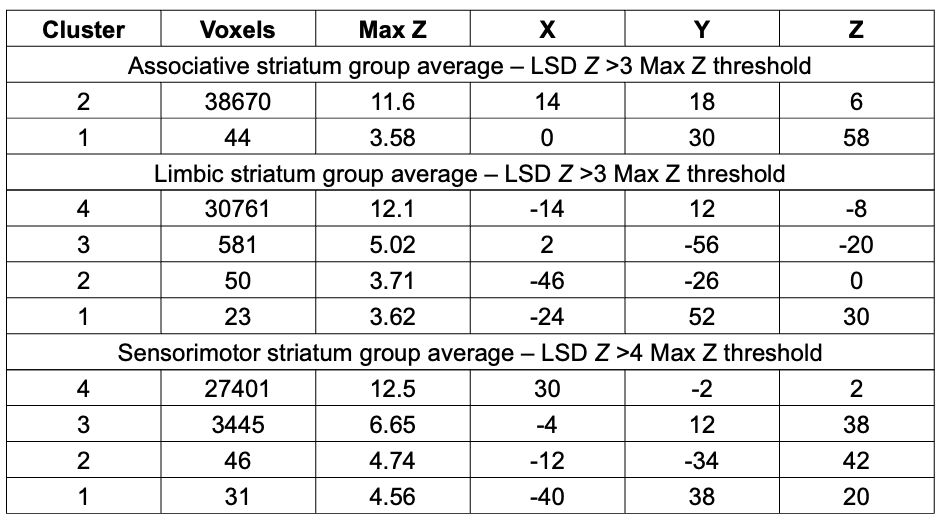
Supplementary Table 1. MNI coordinates for group level LSD results, thresholded at optimal level to define the most clusters.


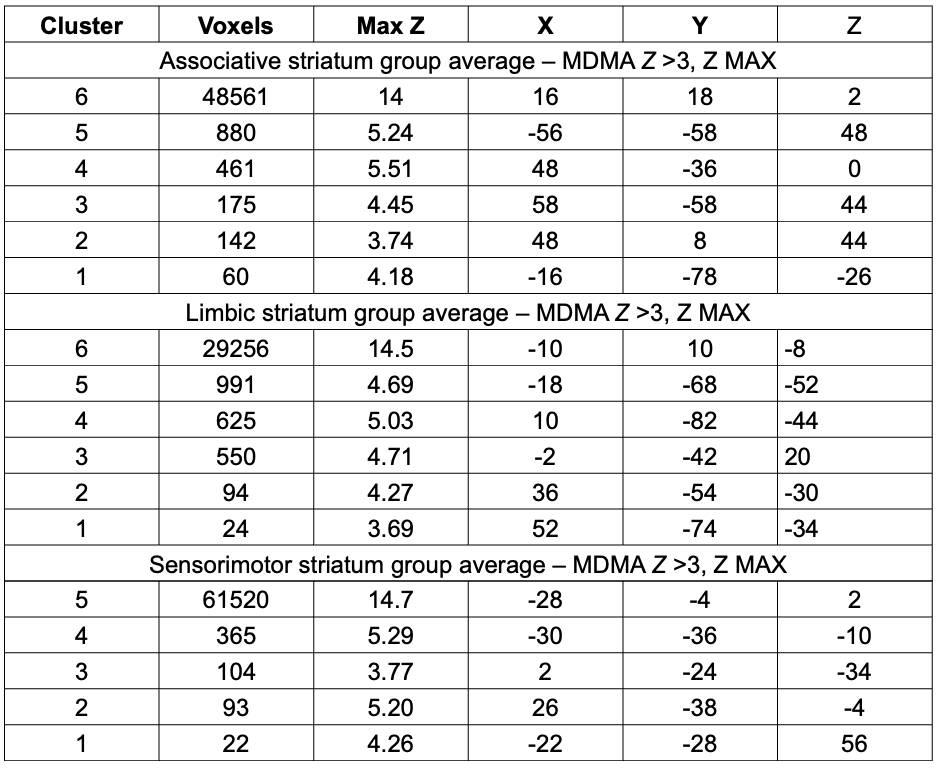


Supplementary Table 2. MNI coordinates for group level MDMA results, thresholded at optimal level to define the most clusters.


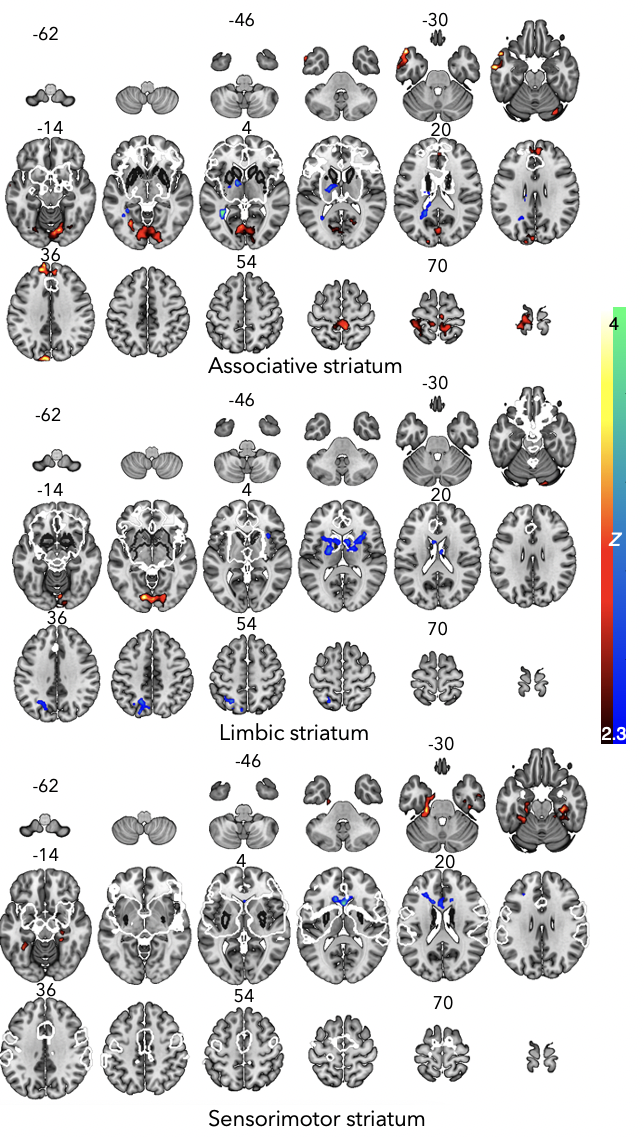


Supplementary Figure 3. Additional slices of acute LSD administration, LSD significantly increased (red/yellow) and decreased (blue/green) connectivity between the A) associative striatum, B) limbic striatum, and C) sensorimotor striatum and areas in the rest of the brain, N=16, results are cluster corrected and thresholded at Z=2.3, p<0.05, original seed regions shown in black and network outline shown in white, MNI slices labelled.


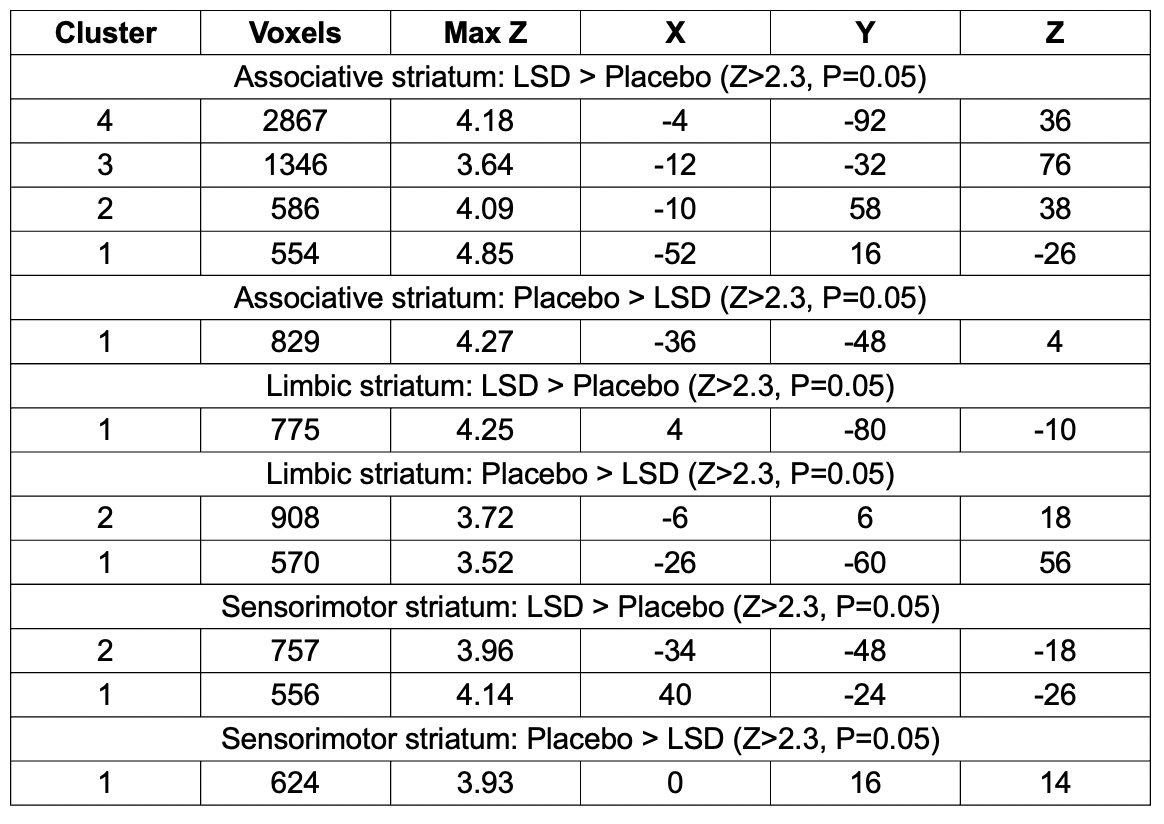
Supplementary Table 3, MNI coordinates showing Z max for each cluster coordinate in acute LSD administration. LSD > Placebo = activation clusters, Placebo > LSD = deactivation clusters.


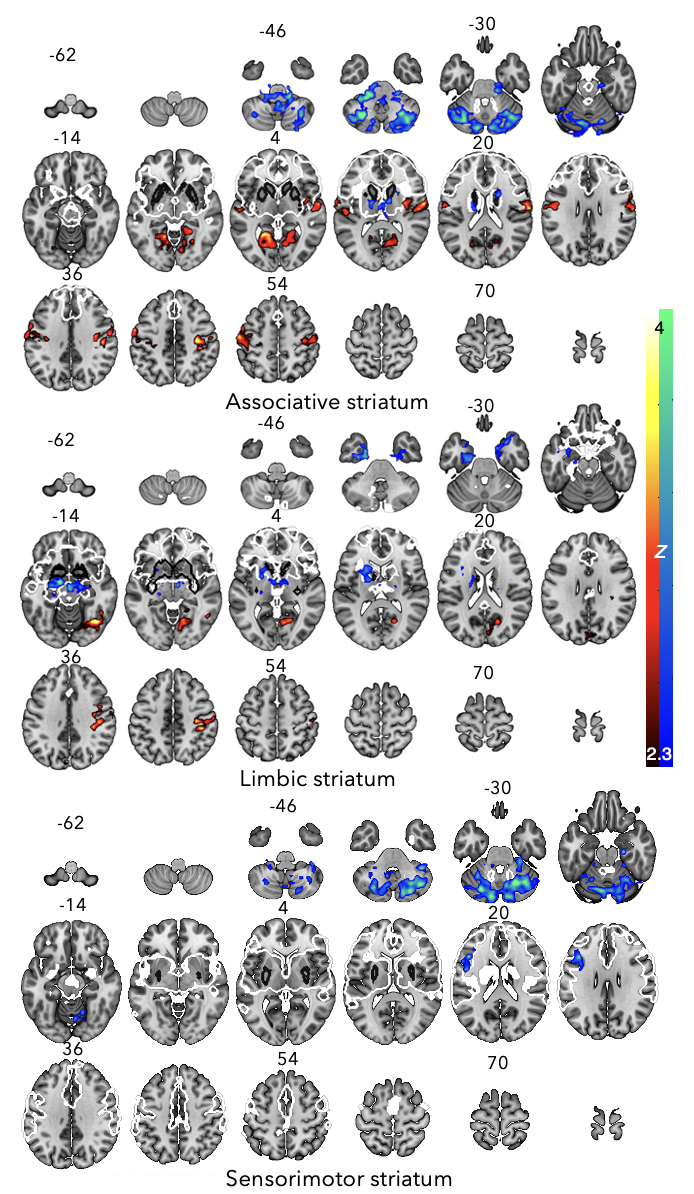
Supplementary Figure 4. Additional slices of acute MDMA administration, MDMA significantly increased (red/yellow) and decreased (blue/green) connectivity between the A) associative striatum, B) limbic striatum, and C) sensorimotor striatum and areas in the rest of the brain, N=22, results are cluster corrected and thresholded at Z=2.3, p<0.05, original seed regions shown in black and network outline shown in white, MNI slices labelled.


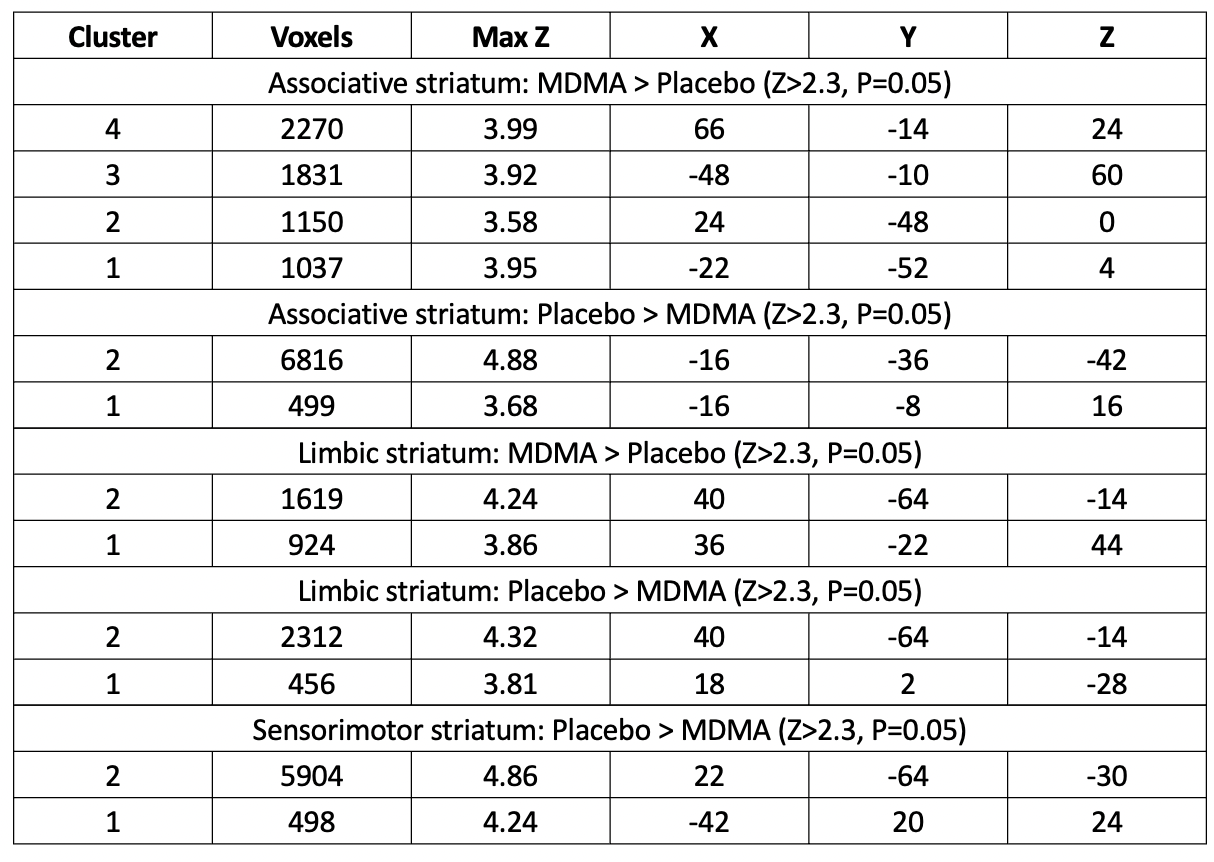


Supplementary Table 4. MNI coordinates showing Z max for each cluster coordinate in acute MDMA administration. MDMA > Placebo = activation clusters, Placebo > MDMA = deactivation clusters.

| LSD |  | Associative striatal network | Limbic striatal network | Sensorimotor striatal network |
| --- | --- | --- | --- | --- |
| Intensity | Pearson's r | -0.013 | 0.068 | 0.108 |
|  | p-value | 0.959 | 0.788 | 0.669 |
| Simple visual imagery | Pearson's r | 0.369 | 0.308 | 0.074 |
|  | p-value | 0.132 | 0.213 | 0.771 |
| Complex visual imagery | Pearson's r | 0.256 | 0.189 | 0.174 |
|  | p-value | 0.305 | 0.453 | 0.49 |
| Emotional Arousal | Pearson's r | -0.119 | 0.165 | 0.11 |
|  | p-value | 0.639 | 0.513 | 0.664 |
| Positive Mood | Pearson's r | 0.15 | 0.398 | 0.135 |
|  | p-value | 0.553 | 0.102 | 0.592 |
| Ego Dissolution | Pearson's r | 0.245 | 0.363 | 0.084 |
|  | p-value | 0.328 | 0.139 | 0.742 |

Supplementary Table 5. Correlation matrix of subjective LSD drug experience and connectivity parameter estimate in the associative, limbic, and sensorimotor striatal network

| MDMA |  | Associative striatal network | Limbic striatal network | Sensorimotor striatal network |
| --- | --- | --- | --- | --- |
| Intensity | Pearson's r | -0.175 | 0.048 | -0.126 |
|  | p-value | 0.425 | 0.828 | 0.568 |
| strange | Pearson's r | 0.139 | 0.333 | 0.235 |
|  | p-value | 0.528 | 0.121 | 0.279 |
| geometric | Pearson's r | 0.059 | 0.125 | 0.121 |
|  | p-value | 0.791 | 0.571 | 0.583 |
| dreamlike | Pearson's r | -0.174 | -0.029 | -0.048 |
|  | p-value | 0.428 | 0.896 | 0.826 |
| imagination | Pearson's r | 0.106 | 0.043 | 0.187 |
|  | p-value | 0.631 | 0.845 | 0.394 |
| time | Pearson's r | 0.191 | 0.437 | 0.342 |
|  | p-value | 0.382 | 0.037 | 0.11 |
| space | Pearson's r | 0.015 | 0.233 | 0.128 |
|  | p-value | 0.945 | 0.285 | 0.561 |
| self | Pearson's r | 0.156 | 0.14 | 0.301 |
|  | p-value | 0.478 | 0.523 | 0.163 |
| muddled | Pearson's r | -0.098 | 0 | -0.048 |
|  | p-value | 0.658 | 0.999 | 0.829 |
| supernatural | Pearson's r | 0.018 | -0.009 | -0.219 |
|  | p-value | 0.936 | 0.969 | 0.316 |
| suspcious | Pearson's r | 0.068 | 0.116 | -0.202 |
|  | p-value | 0.758 | 0.597 | 0.355 |
| merging | Pearson's r | 0.118 | -0.125 | 0.258 |
|  | p-value | 0.59 | 0.569 | 0.234 |
| control | Pearson's r | -0.045 | 0.075 | -0.224 |
|  | p-value | 0.838 | 0.733 | 0.304 |
| mystical | Pearson's r | -0.097 | -0.245 | -0.193 |
|  | p-value | 0.659 | 0.261 | 0.378 |
| wander | Pearson's r | -0.022 | -0.033 | -0.038 |
|  | p-value | 0.92 | 0.881 | 0.862 |
| past | Pearson's r | 0.132 | 0.032 | 0.192 |
|  | p-value | 0.548 | 0.883 | 0.38 |
| peace | Pearson's r | 0.05 | 0.015 | 0.283 |
|  | p-value | 0.822 | 0.945 | 0.19 |
| afraid | Pearson's r | -0.076 | 0.08 | -0.188 |
|  | p-value | 0.731 | 0.715 | 0.391 |
| normal | Pearson's r | 0.025 | 0.003 | 0.109 |
|  | p-value | 0.91 | 0.988 | 0.622 |
| body | Pearson's r | 0.096 | 0.227 | 0.14 |
|  | p-value | 0.664 | 0.298 | 0.524 |
| floating | Pearson's r | 0.093 | -0.029 | 0.177 |
|  | p-value | 0.674 | 0.894 | 0.42 |
| sounds | Pearson's r | 0.373 | 0.423 | 0.138 |
|  | p-value | 0.079 | 0.045 | 0.529 |
| moving | Pearson's r | 0.187 | 0.18 | -0.045 |
|  | p-value | 0.392 | 0.411 | 0.838 |
| energised | Pearson's r | 0.261 | -0.034 | 0.231 |
|  | p-value | 0.229 | 0.876 | 0.289 |
| warped | Pearson's r | 0.02 | 0.078 | -0.013 |
|  | p-value | 0.926 | 0.724 | 0.955 |
| warmth | Pearson's r | 0.193 | 0.059 | 0.17 |
|  | p-value | 0.376 | 0.789 | 0.437 |
| amazing! | Pearson's r | 0.293 | 0.181 | 0.23 |
|  | p-value | 0.175 | 0.408 | 0.291 |
| loved-up | Pearson's r | 0.177 | 0.112 | 0.174 |
|  | p-value | 0.419 | 0.61 | 0.428 |
| sharp | Pearson's r | 0.308 | 0.356 | 0.487 |
|  | p-value | 0.152 | 0.096 | 0.018 |
| super cool | Pearson's r | 0.249 | 0.336 | 0.188 |
|  | p-value | 0.251 | 0.117 | 0.391 |

Supplementary Table 6. Correlation matrix of subjective MDMA drug experience and connectivity parameter estimate in the associative, limbic, and sensorimotor striatal network

# Alternative denoising

The most effective and universally applicable method of mitigating effects of noise in resting-state data is highly debated, with many methods available. Many researchers/labs favour Independent Components Analysis-based methods such as ICA-FIX (Salimi-Khorshidi et al., 2014) or ICA-AROMA (Pruim et al., 2015), while others prefer regressing out signals from tissue-defined regions where non-neural (i.e. ‘noise’) signals are assumed to predominate (e.g. white-matter or cerebrospinal fluid regions). In the present paper we originally used white-matter (WM) and cerebrospinal fluid (CSF) regressors with 24 head-motion parameters, a relatively simple method, similar to that used in a large corpus of previous work (e.g. Carhart-Harris et al., 2017; Comninos et al., 2018; Daws et al., 2022; Ertl et al., 2023, 2024; Roseman et al., 2014; Wall et al., 2019, 2022). An elaboration of this method which is becoming increasing popular with is the anatomical component correction (aCompCor) method (Behzadi et al., 2007; Muschelli et al., 2014). This method applies a principal component analysis (PCA) to time-series from anatomically defined WM and CSF regions; the PCA identifies the WM and CSF components with the highest variance (five from WM, 5 from CSF) and uses these as the nuisance regressors in the model. One disadvantage of aCompCor is it involves inserting a (sometimes large) number of extra regressors into the design matrix, which can reduce the effective degrees of freedom, however it seems to perform favourably when benchmarked against other noise-mitigation strategies (e.g. Hao-Ting Wang et al., 2024). The lack of a true ‘ground truth’ in such benchmarking exercises unfortunately makes objective assessment problematic, however.

As an exploratory procedure, we also used the aCompCor procedure in our analysis as an alternative denoising method. This followed the example of Muschelli et al. (2014) in terms of generating the aCompCor regressors: using WM and CSF regions thresholded at 0.99 in order to generate tight anatomical masks, and then using the top five components from the PCA analyses for both WM and CSF regions to give 10 regressors in total. In addition we followed the example of Parkes et al. (2018) who combined aCompCor regressors with 12 head-motion parameters (original six parameters, plus their temporal derivatives). While this is not strictly comparable to the analyses with WM and CSF regressors (which used a full expanded set of 24 head motion parameters) we felt this was advantageous, partly because of the validation of Parkes et al. (2018), and partly because it kept the total number of regressors more similar (22 for the aCompCor analyses, vs. 26 for the WM+CSF analyses), and thus also approximately harmonised temporal degrees of freedom (in fact, with a slight theoretical advantage for the aCompCor analysis). The WM and CSF masks used for the two analyses also differ, however we felt it was important to replicate the procedure of Muschelli et al. (2014) by using the tight anatomical masks for the aCompCor method.

Images resulting from the basic network definition analyses using the two denoising pipelines are shown in the figures below in a side-by-side format. Results from the two are broadly comparable for the LSD dataset (though with a tendency for the aCompCor analysis to produce larger and more wide-spread clusters). However for the MDMA data, aCompCor has produced network definitinos which appear to be almost global, and cover the entire brain. One possible reason for this is that, as noted by Muschelli et al. (2014), aCompCor regressors tend to include more of the global signal than ‘standard’ WM and CSF regressors. As noted above, assessing denoising effectiveness can be problematic because of the lack of a ‘ground truth’ however, in these cases, it seems clear that the WM and CSF analyses have produced networks which are a) more anatomically plausible, b) more consistent across the two datasets, b) more consistent with previous similar work using these seed regions (e.g. Wall et al., 2022), and d) generally more conservative in their definitions. For these reasons we opted to persist with our standard approach of using WM and CSF regressors for the main analyses presented in the paper, while providing the images below for the purposes of full transparency. See supplementary figures 5 and 6 below.


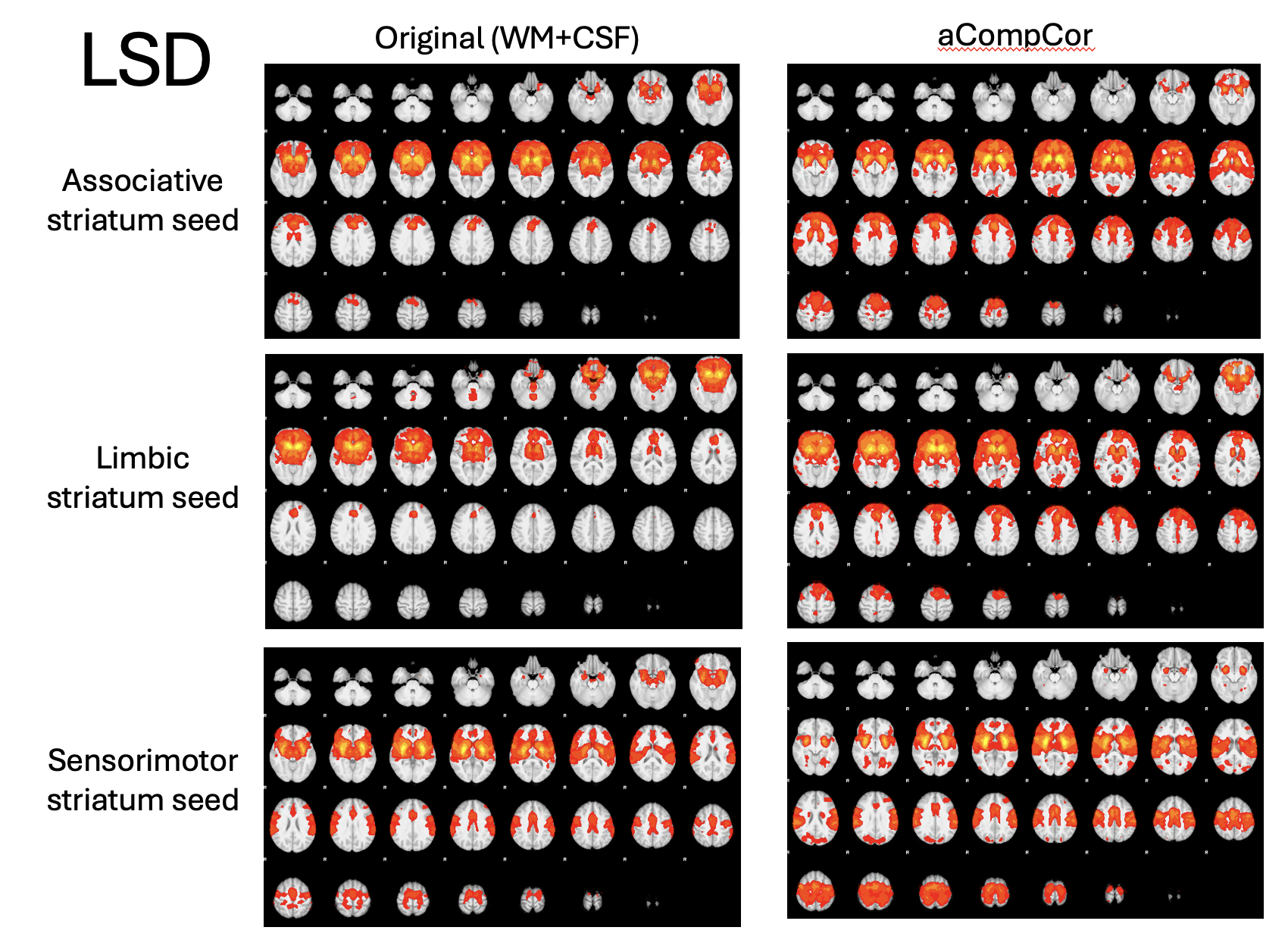


Supplementary Figure 5. Comparison of networks from group average models with the Associative, Limbic, and Sensorimotor striatal seeds with the denoising method used in the present paper (Original WM+CSF) and the alternative aCompCor method in the LSD cohort. Thresholded at Z=2.3, p < 0.05 (cluster-corrected for multiple comparisons).


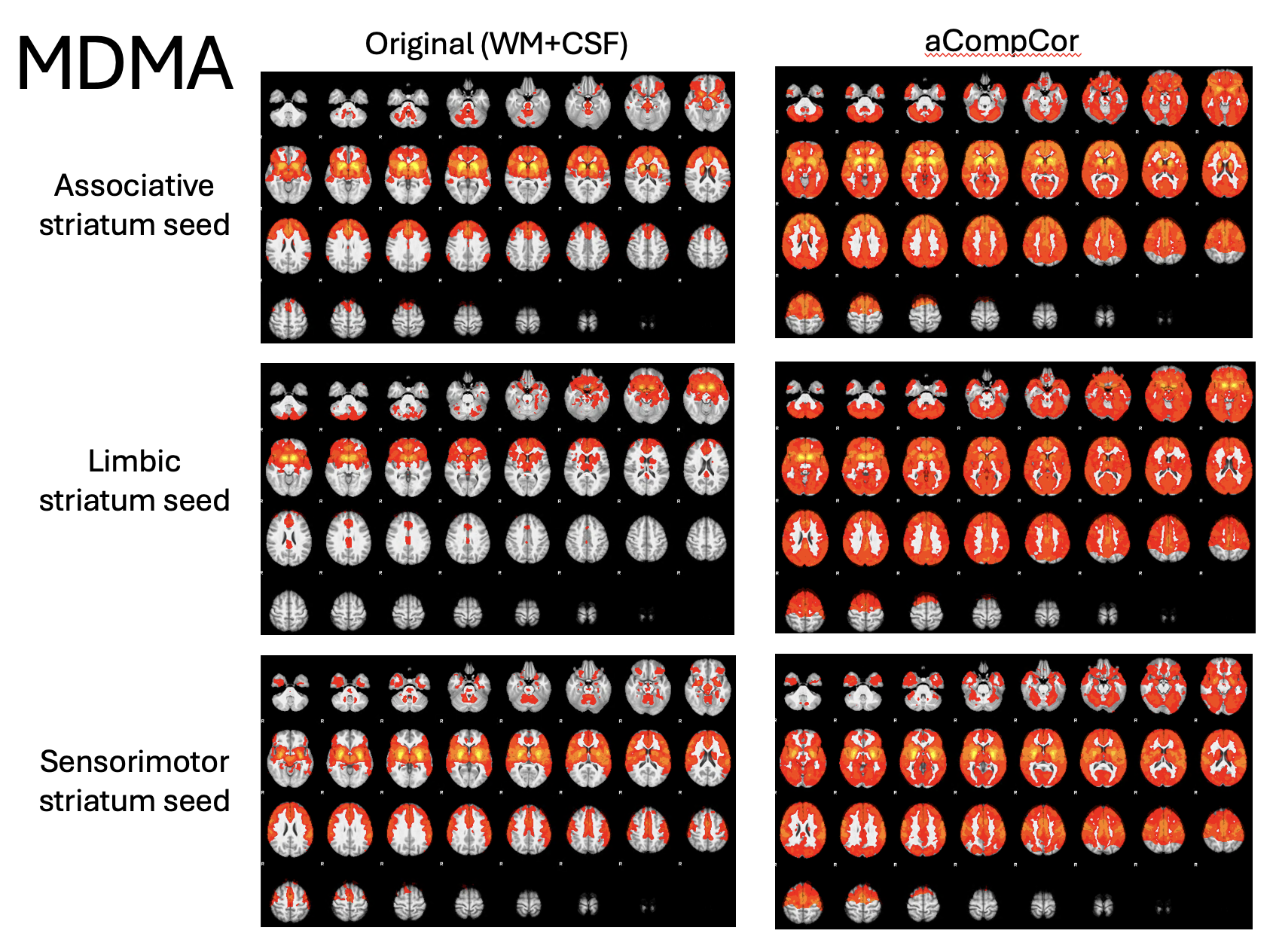


Supplementary Figure 6. Comparison of networks from group average models with the Associative, Limbic, and Sensorimotor striatal seeds with the denoising method used in the present paper (Original WM+CSF) and the alternative aCompCor method in the MDMA cohort. Thresholded at Z=2.3, p < 0.05 (cluster-corrected for multiple comparisons).
